# When the Going Gets Tough … we Discuss and Debate!

**DOI:** 10.1177/14550725261506883

**Published:** 2026-11-12

**Authors:** Matilda Hellman

**Affiliations:** 1 Department of Sociology, 225313 Uppsala University Department of Sociology, Uppsala, Sweden; 2 154260 University of Helsinki Faculty of Social Sciences, Helsinki, Finland

**Keywords:** Addiction Severity Index, cannabis harm, cannabis policy, qualitative focus groups, The Nordic Association for Research on Alcohol and drugs (NARAD)

This final issue of *Nordic Studies on Alcohol and Drugs* (*NAD*) for 2026 is a vivid manifestation of a dynamic, scientifically substantial, and socially relevant field of research. It is, in short, packed with discussions that our readers will not want to miss. We look forward to further contributions and reactions, and hope that this will lead to new ideas and future collaborative projects.

Three extensive discussions are initiated. The first concerns how we should understand and evaluate the extent of the harm caused by cannabis.

Fischer et al. (2026) raise the question of how scientific questions and foci shape what we mean by cannabis-related harms. The authors argue that evaluations of cannabis legalization in Canada may have overlooked an important public health issue: whether legalization has affected the use of alcohol and tobacco, as well as the harms associated with them. Since alcohol and tobacco contribute to significantly higher levels of morbidity and mortality at the population level than cannabis itself, such effects could substantially alter the overall assessment of the consequences of cannabis legalization. This is, indeed, a reasonable question to ask. Their text also raises broader questions regarding causality and how we, from an ontological perspective, understand “harm.”

In their reflections on the initiative by Fischer and colleagues, Järvelin and Hupli (2026) broaden the perspective and introduce another level of analysis. While they agree about the importance of studying the relationship between cannabis legalization and the use of alcohol and tobacco, they note that Fischer and colleagues primarily address the issue at the population level, based on prevalence data. Järvelin and Hupli argue that there are other significant aspects to consider that could provide a clearer picture of the situation. Specifically, they highlight use practices and consumption cultures as areas of inquiry that may offer further insight into this matter. If we are to examine alcohol and tobacco use in the context of the consequences of cannabis legalization, we must understand how these substances are linked, in terms of whether they are being added to, mixed with, or substituted for one another in actual patterns of use.

Jacob Manthey and Daniel Kotz (2026) take a different approach in his commentary: they examine Fischer's problem formulation through the lens of the German cannabis reform and how it can be evaluated. The authors describe an emerging divergence in research findings: cannabis-related crime appears to be decreasing significantly, certain cannabis-related health issues seem to have risen slightly, while the prevalence of cannabis use has not shown a clear increase at the population level. From this discussion, we can conclude that more data from various countries, implementing different forms of legalization, are required to address this issue convincingly from such an integrated perspective. Furthermore, one must not overlook the point also raised by Järvelin and Hupli: aggregate data cannot be interpreted, or even properly designed, without a genuine understanding of the underlying logics of use as they manifest at the micro-level.

The second debate initiated in this issue expresses scepticism regarding the Swedish National Board of Health and Welfare's (Socialstyrelsen) strong recommendation of the Addiction Severity Index (ASI) within Swedish addiction treatment. Is such a strong recommendation for its use actually supported by evidence?

Alexander Björk (2026a) argues that evaluations of this instrument fail to provide convincing evidence that it leads to better decision-making or improved outcomes for clients. In fact, diagnostic intervention studies for the ASI are lacking. While it is true that the instrument can measure certain aspects quite effectively, this is not the same as measuring outcomes. Have practical conclusions perhaps been drawn prematurely? Björk finds the National Board of Health and Welfare's justification for using the ASI unconvincing; for one thing, the underlying data is neither representative of the Swedish population nor of the specific clients for whom the ASI is used. Furthermore, the justification for its implementation relies on the argument of already well-developed, standardized manuals and guidelines for practitioners – but this does not constitute robust evidence of positive outcomes, as Björk points out. He speculates that the tool's strong recommendation may be due to a form of “path dependency” within the system. Substantial resources – technological, financial, professional, and organizational – have been invested, creating pressure to proceed with implementation. Additionally, the National Board of Health and Welfare occupies a dual role: it is both the owner of the ASI and the entity that produces the guidelines for its use.

Three responses to Björk shed light on the issue from different, partly complementary perspectives. Jørgen Bramness (2026) largely agrees with much of Björk's critique, but points out that the tool has gained popularity precisely because it recognizes that substance abuse problems often spill over into – and affect – various areas of life (such as employment, finances, physical health, and family). However, the instrument is undeniably costly to implement; the ASI requires training and is time-consuming. Could other instruments perhaps provide a similar overview more simply and at a lower cost? Ultimately, Bramness lands a nuanced stance: perhaps the ASI should not be abandoned for these reasons, especially considering the resources that have already been invested.

The second commentary on Björk's text comes from Katja Kuusisto (2026), who turns attention to what happens after the assessment. While an instrument may certainly be useful for structuring a conversation or highlighting problems, this does not automatically mean that the client receives better support. Which needs are brought to light, and does the client have the opportunity to correct or supplement the picture presented? One might also ask how this information influences professional decision-making. If documentation practices shape how clients’ situations are described and understood, we should turn our attention to what these practices overlook and what they emphasize.

In the third commentary, Anette Nielsen (2026) highlights the value of the ASI as a multi-functional tool: it facilitates assessment and planning, enables follow-up and the reporting of outcomes, and provides research data and composite scales. As a standardization tool, it helps build a comprehensive overview of a given situation. It allows for the comparison of client groups and treatment outcomes, while also providing a baseline for research. For an established international instrument like the ASI to be abandoned, any replacement would have to be significantly better.

The debate surrounding the ASI includes many other perspectives that are worth reading. Furthermore, Björk (2026b) addresses the three contributions in a brief commentary.

The third “For Debate” in this issue of *NAD* addresses focus groups as a method with functions that extend beyond mere data collection. Michael Egerer (2026) weighs in on the contribution of focus groups, arguing that they also serve as small-scale arenas for civic education; consequently, they are better able to capture emerging attitudes and opinions than survey-based research. The differences in the scientific contributions of surveys versus focus groups became particularly evident in two parallel studies concerning the Finnish gambling monopoly, Veikkaus (Egerer et al., 2018; Salonen et al., 2017), which were conducted around the same time. When the researchers compared the focus groups with the parallel survey, they found that the surveys indicated relatively strong support for the existing system, whereas focus group participants expressed significantly more criticism regarding gambling advertising, the accessibility of slot machines, and the design of gambling policy. Egerer et al. (2018) consider the capacity of focus groups to foster self-reflection and peer-to-peer learning among participants. In the case under discussion, as suggested by Egerer et al. (2018), they also functioned as a form of simulated civic dialogue or as learning environments.

This issue also features new peer-reviewed research in a topical research article by Vaarankorpi et al. (2026), which explores online material from the Finnish AddictionLink information service, with the aim of describing the types of drugs and drug-interaction questions being submitted.

We are also pleased to include more cultural critique in the journal. In this issue, Paula Rautoja et al. (2026) examine the film *Cambodian Beer Dreams*, using it as a starting point to illustrate a broader field of research concerning the power of the alcohol industry and the commercial determinants of health. And if you missed the short analysis of Stephen King in our previous issue (Marquez-Romero, 2026), please do take a look!

## A new Association

It is a pleasure to conclude the year 2026 with such a debate-rich issue, especially as changes are on the horizon: starting in January 2027, ownership of *Nordic Studies on Alcohol and Drugs* (*NAD*) will be transferred from the Nordic Welfare Centre (NVC) to a newly founded association, the Nordic Association for Research on Alcohol and Drugs (NARAD). At the time of writing, we do not yet know what our budget or staffing situation will be, among other details. For the time being, the journal will continue to be published by SAGE.

The association is a welcome addition to a Nordic research field that has been substantially weakened over the past decade. Across the Nordic countries, the centres and institutes responsible for monitoring and producing knowledge on alcohol, drugs, gambling, and other addictive behaviours have shrunk. This autumn, the board of the Centre for Social Research on Alcohol and Drugs (SoRAD) at Stockholm University has initiated the closure of the centre. This decision stems from limited prospects of effectively and fully meeting the objectives of being a national leader and internationally prominent in the field. At the same time, high-quality and socially relevant research within SoRAD's area of focus continues to be conducted within the Department of Public Health.

At the Norwegian Institute of Public Health (FHI), a process has been initiated to lay off several employees and alcohol and drug research is likely to be affected by the organizational change. At the Centre for Alcohol and Drug Research (CRF) at Aarhus University, one-third of the centre was dismantled a few years ago. In total, there are around 30 people at the CRF conducting research across a variety of subfields, with several projects funded by external grants. Overall, this represents a reduction of approximately ten people compared to a couple of years ago.

At the Finnish Institute for Health and Welfare (THL), the team concerned with national research on all aspects of alcohol and drug, on the one hand, and the one concerned with the major share of Finnish gambling research, on the other, each consist of around 20 people, with 50% of the alcohol and drug researchers receiving external funding and most performing other tasks at THL as well.

But research, investigation, and collaboration are also taking place through various national networkds, through individual Nordic initiatives, involving researchers scattered across universities and diverse institutes, as well as officials and members of civil society. Nevertheless, it is no exaggeration to say that the field has become considerably weaker over the last couple of years.

The great irony is that humanity has arguably never been surrounded by such a proliferation of potentially addictive behaviours. Alongside alcohol, drugs, doping, nicotine, and gambling, we are increasingly confronted with problematic relationships to screens, sugar, caffeine, news, social media, shopping, and other commodities and activities. These problems are intensive, dramatic and complex, and they are becoming more difficult to grasp and entangled with health issues.

The conditions under which we understand these developments are changing also in view of new kinds of misinformation, knowledge denial and ignorance. We are entering a period in which a person's ability to navigate society will depend not only on economic resources, but also on their capacity to critically assess information, understand where it comes from, and judge what value and credibility it has. This creates an excellent opportunity for commercial forces to profit from our addictive behaviours.

A small research field such as the Nordic social-scientific addiction research community, or the alcohol and drug research community as it has traditionally been called, is not in a position to confront these global forces on equal terms. But it can represent something important: our countries’ commitment to developing societal responses grounded in evidence-based knowledge.

It can also represent a willingness to collaborate around the principles that have been important to the Nordic societies and that remain visible in how substance use and substance use problems are addressed. The Nordic values at stake can be understood as a continuity of the priorities that the societies have placed on education, public health, and social care. An essential part of this commitment is, undoubtedly, the monitoring of and research into substance use and addiction-related problems in our populations.

This is why maintaining and strengthening the Nordic research infrastructure matters. And this is why the establishment of NARAD, and the continued existence of *NAD* as a Nordic research journal, comes at a particularly important moment.

To end on a nostalgic note, two texts have lately reminded me of the importance of being able to draw generalizing conclusions and plug into larger discussions:

In Hans L. Zetterbergs piece from 1966 on the institutionalization of sociology and the social sciences in the Nordic countries, he specifically mentions the importance of research institutes established outside the universities (Zetterberg, 1966). Examples include the Institute for Social Research (Institutt for samfunnsforskning) in Oslo, the Danish National Institute of Social Research (Socialforskningsinstituttet) in Copenhagen, and the Alcohol Research Institute in Helsinki. These institutions were part of a broader post-war expansion of social science expertise, characterized by a growing emphasis on empirical research (often survey methods) and the systematic production of knowledge to inform public policy and make society more amenable to scientific understanding and governance. During the 1960s the Finnish institute's remit broadened towards more fundamental investigations of alcohol's role in society, beyond the evaluation of individual control measures. In hindsight, Zetterberg was right in his claim: the institutes would indeed become part of establishing and developing of Nordic sociology over the following decades.

Why the alcohol and drug research field has played and is still playing such a big role in the development of social sciences is not only because of the fact that millions of Nordic citizens are affected by these questions. In a book chapter from 2007 by Olavi Kaukonens och Kerstin Stenius, the authors remind us that while the substance use treatment field may, in economic terms, be a relatively small part of the welfare state, it is well suited and useful for the study of changes in the normative foundation of the welfare state (Kaukonen & Stenius, 2007). Kaukonen and Stenius are concerned that professionalization and technocratization (my term) make it increasingly difficult to openly debate questions of equality, solidarity and society's responsibility. All the more reason to be pleased that we are still discussing and debating and that the discussions take on more general questions, such as the ones in this issue.

It has been vital that our governments have understood the importance of supporting research in this field; yet, it is also thanks to the fact that we have continued to engage in discussion, maintain an interest in one another, and pose difficult questions that our field of research has nonetheless become something of a success story.
